# Chromodomain Helicase Binding Protein 8 (Chd8) Is a Novel A-Kinase Anchoring Protein Expressed during Rat Cardiac Development

**DOI:** 10.1371/journal.pone.0046316

**Published:** 2012-10-10

**Authors:** Maureen O. Shanks, Linda M. Lund, Sabrina Manni, Mary Russell, Joseph R. H. Mauban, Meredith Bond

**Affiliations:** 1 Department of Physiology, University of Maryland Baltimore, Baltimore, Maryland, United States of America; 2 Department of Biochemistry, University of Maryland Baltimore, Baltimore, Maryland, United States of America; 3 Department of Medicine, Clinical Immunology and Hematology Branches, University of Padova, Padova, Italy; 4 Venetian Institute of Molecular Medicine (VIMM), Padova, Italy; 5 Department of Biological Sciences, Trumbull Campus, Kent State University, Warren, Ohio, United States of America; 6 College of Sciences and Health Professions, Cleveland State University, Cleveland, Ohio, United States of America; University of Oslo, Norway

## Abstract

A-kinase anchoring proteins (AKAPs) bind the regulatory subunits of protein kinase A (PKA) and localize the holoenzyme to discrete signaling microdomains in multiple subcellular compartments. Despite emerging evidence for a nuclear pool of PKA that rapidly responds to activation of the PKA signaling cascade, only a few AKAPs have been identified that localize to the nucleus. Here we show a PKA-binding domain in the amino terminus of Chd8, and demonstrate subcellular colocalization of Chd8 with RII. RII overlay and immunoprecipitation assays demonstrate binding between Chd8-S and RIIα. Binding is abrogated upon dephosphorylation of RIIα. By immunofluorescence, we identified nuclear and perinuclear pools of Chd8 in HeLa cells and rat neonatal cardiomyocytes. We also show high levels of Chd8 mRNA in RNA extracted from post-natal rat hearts. These data add Chd8 to the short list of known nuclear AKAPs, and implicate a function for Chd8 in post-natal rat cardiac development.

## Introduction

Stimulation of G-protein coupled receptors results in elevated amounts of the second messenger molecule cAMP, leading to the activation of protein kinase A (PKA). PKA is a holoenzyme composed of two regulatory (RI or RII) and two catalytic (C) subunits. Activated PKA phosphorylates key substrates (reviewed in [1]). A-kinase anchoring proteins (AKAPs) anchor PKA, by means of its R subunit dimer, to subcellular structures [2], forming microdomains (recently reviewed in [3]–[5]). This binding confers subcellular localization of PKA, and contributes to PKA specificity and the rapid and effective modulation of PKA-dependent signaling [6].

More than 70 AKAPs have been described to date in multiple cell types and cellular compartments. [1], [3], [7] Defects in AKAP anchoring or expression have been linked to pathologies such as cardiac arrhythmia [8]–[11], hypertrophy [12]–[14], and the progression to heart failure [15], [15]–[18]. Our studies, and those of others, have demonstrated that PKA target phosphorylation is decreased in failing hearts. [15], [19], [20] The importance of proper PKA signaling in failing myocardium is also linked to transcription, as treatment of patients by use of β-blockers reverses the fetal gene switch between α-myosin heavy chain and β-myosin heavy chain. [21]–[23] Taken together, the scaffolds formed by AKAPs represent a powerful mechanism for mediating PKA signaling in the cell, and impaired AKAP:PKA interaction has serious implications for development of cardiac disease.

AKAPs are a functionally diverse family with little sequence similarity, except that they share a characteristic amphipathic α-helical domain that is approximately 14–18 amino acids (aa) in length. [24] This helix has a hydrophobic face that fits in a groove formed by the amino-termini of the R dimer. [24]–[26] In binding PKA as well as PKA substrates and regulators, AKAPs create a scaffold to effectively localize and modulate signaling through PKA. Given the importance of AKAPs in cardiac function, we used a T7 phage display assay to identify PKA binding proteins. [27] When we screened a cDNA library derived from human heart, we isolated multiple clones of chromodomain helicase binding protein 8 (Chd8), suggesting that Chd8 could also act as an AKAP.

**Figure 1:**
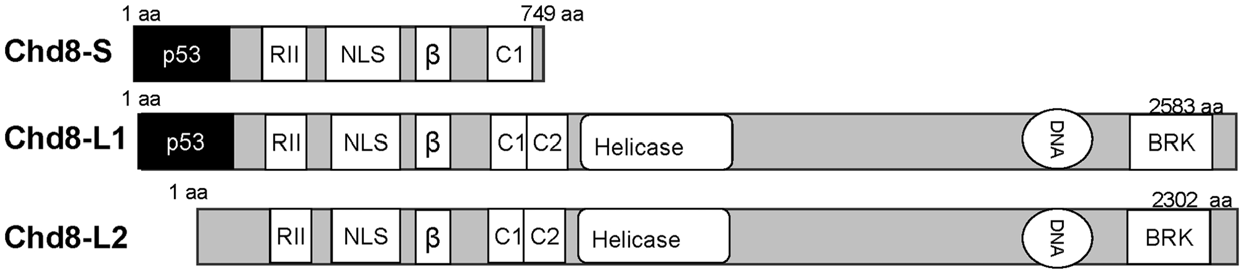
Three isoforms have been identified for Chd8. Chd8-S arises from passage of transcription through the end of exon 9 into intron 10, where it terminates. [32], [34] Chd8-S contains a single chromodomain (C1). Two longer isoforms contain the tandem chromodomains (C1 and C2) and helicase domain characteristic to Chd proteins [91], [92]. Chd8-L1 and Chd8-L2 result from two different start sites for transcription [32], with the Chd8-L1 transcript encoding an amino terminus extension that encompasses a p53 binding domain [36]. All three isoforms contain a series of five nuclear localization signals (NLS) [38] and a β-catenin binding domain (β) [31], [37], [39] that is also required for histone H1 [35] binding [35] and STAT3 [28] binding. Chd8-L1 and Chd8-L2 also contain a pair of BRK domains, which mediate chromatin interaction via the histones and is required for interaction with CTCF [29]. All three isoforms contain the AKAP domain (RII) characterized in this study.

In studies conducted to date, Chd8 has been primarily characterized as a nuclear protein [28] that regulates chromatin dynamics [29]–[31], transcription [30], [32], [33], and cell survival [34]–[36]. Chd8 was first identified as a 749aa nuclear protein named “duplin,” [37], [38] and found to inhibit the Wnt signaling pathway. [37] In humans, Chd8 predominantly exists in two larger isoforms, Chd8-L1 (2582aa) and Chd8-L2 (2301aa). [29], [32], [33] (***Figure 1***) Chd8 contains binding sites for and negatively regulates β-catenin [31], [35], [37], [38] and p53 [36] by means of its ability to bind histone H1. Loss of Chd8 in knockout mice resulted in an embryonic lethal phenotype at embryonic day (E) 8.5 resulting from increased p53-dependent apoptosis. [36], [39] Double knockout of Chd8 and p53 extended embryo survival to E10.5, at which point embryos failed to form mesoderm and exhibited massive hemorrhaging characteristic of cardiovascular defects. [36] Chd8 binds other components of transcription, including di−/trimethylated lysine 4 on the histone H3 subunit (H3K4) [30], [32], [40], RNA polymerase II [32], and members of the Mixed Lineage Leukemia (MLL) complex, WRD5, ASH2L, and RbBP5 [40]. Considering the diversity of its binding partners, Chd8 is likely a component of the scaffold of several large protein complexes, each with a distinct function [40].

Given the evidence for a nuclear microdomain of PKA [41], [42], we hypothesized that Chd8 is a novel nuclear AKAP. Also, as Chd8 has largely been studied as a nuclear protein and in the context of development [37]–[39], we investigated subcellular localization and expression of Chd8 during cardiac development and in non-cardiac cells. Our findings demonstrate a novel localization of Chd8 to a discrete perinuclear microdomain, as well as to the nucleus, and define a new link between PKA-dependent signaling and proteins responsible for chromatin remodeling.

## Materials and Methods

### Ethics Statement

All animal studies were conducted in compliance with the Animal Welfare Act, Public Health Service Policy on Humane care and Use of Laboratory Animals, and in compliance with the *Guide for the Care and Use of Laboratory Animals,* published by the National Institutes of Health (NIH publication No. 85–23, revised 1996). All animal work was performed under protocols approved by the Institutional Animal Care and Use Committee of the University of Maryland, School of Medicine.

### Antibodies, Reagents

Commercial Chd8 antibodies were obtained from Bethyl Laboratories and used for Western blotting and immunoprecipitation (Bethyl Laboratories, Montgomery, TX), and immunofluorescence (Bethyl). Anti-myc-epitope (Cell Signaling Technology, Danvers, Massachusetts), RIIα/β (EMD Millipore, Billerica, Massachusettes), RIIα (BD, Franklin Lakes, New Jersey), RIIß (BD), and anti-Golgi apparatus (EMD) antibodies were used for western blotting of immunoprecipitates or immunofluorescence, as described. GAPDH (Life Technologies/Ambion, Grand Island, New York) was used for loading control.

### Cell Culture

Pregnant Sprague-Dawley female rats were ordered from Harlan Labs (Frederick, Maryland). Primary neonatal cardiomycytes (NCMs) were harvested from pups at post-natal day 1 and cultured as previously described. [27], [43] CHO cells (American Type Culture Collection, Manassas, VA) were cultured in Ham’s F12 media with 10% FBS. HeLa cells (ATCC) and HEK cells (ATCC) were cultured in DMEM media (high glucose) with 10% FBS. All transfections were carried out with Lipofectamine-2000 (Life Technologies/Invitrogen).

### Plasmids

A plasmid for the ‘duplin’ isoform of Chd8 (which we refer to as Chd8-S) was kindly provided by Dr Akira Kikuchi, Hiroshima University, Japan. The QuikChange XL Site-Directed Mutagenesis Kit (Agilent/Stratagene, Santa Clara, California) was used to introduce point mutations of key constructs, according to the manufacturer instructions. RIIα, RIIα-SA, and RIIα-SD mutants were created as described [20] and cloned into peGFP-C1 (Clontech Laboratories, Mountain View, California), in which a CFP was substituted for GFP, for creation of CHO cell lines.

### Phage Display Screening

Phage display screening was performed using a human heart cDNA library as previously described. [27] Briefly, a 96-well dish was coated with recombinant RIIα purified from *E. coli* expressing RIIα-pET11d. 106 clones from a T7-select Phage Display Library (EMD) specific for human heart cDNA were screened. Phage-specific primers were then used for PCR amplification of RII binding peptides, which were sequenced (DNA Sequencing Core Facility, Lerner Research Institute, Cleveland Clinic Foundation) and analyzed with Lasergene software (DNASTAR) and BLAST programs (NCBI, National Institutes of Health). Three clones were isolated and identified by BLASTn search as Chd8.

### Western Blotting

For protein extraction of transfected cells, cells were lysed 48 hours after transfection with M-PER Mammalian Protein Extraction Reagent (Thermo Scientific, Rockford, Illinois) with protease inhibitor cocktail (Sigma-Aldritch). NCMs were harvested for protein extraction four days after isolation using a buffer containing M-PER, 40 mM EDTA, 300 mM NaCl, and protease inhibitor cocktail (Sigma-Aldritch), as described. [27] Micro-BCA was used to determine protein concentration (Thermo Scientific). Lysate was boiled with 4×SDS loading buffer (with DTT), separated by SDS-PAGE, and transferred to PVDF. Western blots used 50 micrograms of total protein per lane, unless noted. The blot was blocked with 5% milk-Tween solution. Blots were incubated with SuperSignal West Pico Chemiluminescent Substrate (Thermo Scientific), and positive bands detected by chemiluminescence. Primary antibodies were used at a dilution of 1∶2000, and secondary antibodies at a concentration of 1∶10,000, except where otherwise noted in the figure legend.

### RII Overlay

PCR primers were used to generate cDNA encoding the RII binding site of rat Chd8 (Chd8^RII^). This cDNA was cloned into the pTrcHis2/TOPO vector (Invitrogen). Colonies were selected based on antibiotic resistance and sequenced by the Genomics Core Facility (University of Maryland, Baltimore). Bacteria were grown to an OD600 value of 0.6, and isopropyl-beta-D-thiogalactopyranoside (IPTG) was added to induce expression according to manufacturer’s instructions. A control vector containing LacZ was transformed and used to verify expression. Samples were taken hourly, prepared with 1× Laemli loading buffer with β-mercaptoethanol, separated by electrophoresis and transferred to nitrocellulose for Western blotting. For RIIα overlays, 5 mL of bacterial culture suspended in LB was collected at hour 3, spun down, and lysed in 1× SDS loading buffer. Bacterial lysate separated on a 12% Tris-HCl gel and transferred to nitrocellulose membrane. The control expression protein (LacZ) as a negative control. Purified recombinant RIIα (5 µg/mL in 10% BSA/TBS), was incubated alone, or with either 50 uM Ht31 or Ht31P peptides (generated by Biopolymer Core, University of Maryland, Baltimore). After blocking for 1 hour, separate blots were incubated overnight with the RIIα solutions. Each blot was washed thoroughly and developed using antibody against RIIα/β (EMD).

### Co-immunoprecipitation

Immunoprecipitations of tagged protein constructs were conducted with the myc-tag co-immunoprecipitation kit (Thermo Scientific) according to manufacturer’s instructions.

### Immunostaining and Inverted Fluorescent Microscopy

Cells were grown on glass coverslips and fixed with 4% paraformaldehyde in PBS for 10 minutes at room temperature, then permeabilized with 0.1% Triton X/PBS for 10 minutes. Cells were blocked with 3% BSA/PBS, and then incubated with primary antibody at the following dilutions: myc antibody (CST, 1∶250), Chd8 (Bethyl, 1∶200 or 1∶50), RIIα/β (EMD, 1∶80), Golgi apparatus (EMD, 1∶100), α-actinin (Sigma-Aldritch, 1∶250). For Figure S4, cells were immunolabeled with Chd8-Sigma, a polyclonal antibody purified from anti-sera from New Zealand White rabbits inoculated with 77–90aa of Chd8 (Sigma-Aldrich Genosys, St Louis, Missouri). Sera was purified, eluted from the purification column, and used for immunofluorescence at a dilution of 1∶250 (HeLa) or 1∶150 (primary rat neonatal cardiac cells). Cells were washed for 1 hour with 1% BSA/PBS, incubated with Alexafluor conjugated antibodies (Life Technologies/Invitrogen, Grand Island, New York) as indicated at 1∶500, and washed again. Coverslips were mounted and nuclei were stained using Vectashield mounting medium with DAPI (Vector Laboratories, Burlingam, California). All antibody incubations occurred for 1 hour at room temperature, except for myc and Chd8-Sigma, which occurred at 4°C overnight. Immunostaining was visualized with a Nikon TE2000-U inverted fluorescent microscope (Nikon Instruments, Melville, New York) and images were obtained with a Spot digital camera (Diagnostic Instruments, Inc., Sterling Heights, Michigan) with Spot Advanced 4.0.2 software.

### Confocal Microscopy

A Zeiss 5 Live slit scanning confocal microscope was used to image HeLa cells that were colabeled with antibodies for Golgi apparatus and Chd8. Imaging was performed with a 63X, 1.4 NA oil immersion objective, and data acquisition was set at .21 µm/pixel, 12 bit. The Alexafluor goat anti-mouse 568 antibody used to detect Golgi signal was excited at 561 nm and emission was long pass filtered at 575 nm. Excitation of Chd8 signal, detected by Alexafluor goat anti-rabbit 488 was at 489 nm and emission was bandpass filtered at 495–555 nm.

### Taqman Quantitative PCR and Analysis

Rat hearts were harvested for RNA extraction with the Quick Prep Total RNA Extraction kit (GE Life Sciences, Piscataway, New Jersey) at embryonic days (16 and 19), post natal days (1, 3, 7, and 21), and six months. RNA was converted into cDNA with the iScript kit (Bio-Rad, Hercules, California). 20 ng of cDNA was used per assay. Taqman probes for Chd8 (Rn00576005_m1, designated Probe 1, and Rn01414467_m1, designated Probe 2), RIIα (Rn00709403_m1), and GAPDH (Rn 99999916_s1) were obtained from Applied Biosystems, and used in conjunction with Master Mix (Applied Biosystems, Carlsbad, California) as described for PCR amplification with a 7900HT Thermal Cycler (Applied Biosystems), as described. [44] Each sample was assayed in triplicate, and the average of the three values was normalized to GAPDH (internal reference). Relative quantitation of mRNA was calculated using 2^−ΔΔCt^ method, as described elsewhere [45], with embryonic day 16 set to “1”.

## Results

### Identification of Chd8 as a Novel Binding Partner for RIIα

The T7 phage display assay was used to screen a human heart cDNA expression library to identify novel cardiac AKAPs. This approach identified several known RIIα binding proteins that served as positive controls and verified our method. These included RIIα [46] and two known cardiac AKAPs, mAKAP [47] and AKAP-Lbc [12], [48]. We isolated clones that contained fragments of seven previously unidentified RIIα binding proteins. One of those proteins, synemin, is an intermediate filament protein that we characterized as a novel AKAP that associates with the Z-discs and cell junctions in cardiac myocytes [27], [44].

Among the proteins we identified as binding partners for RII, three clones contained peptide fragments of Chd8. Using BLAST analysis of the peptide fragments, we found that the three clones overlapped with the amino terminus of Chd8 and encoded 391–545aa of the Chd8-L1 isoform. This sequence is present in all three isoforms of Chd8 and is located immediately upstream of a nuclear localization signal (NLS). (***Figure 1***) Because of the known role for Chd8 in regulating transcription and cell survival, we focused our investigations on the potential role of Chd8 as an AKAP.

**Figure 2:**
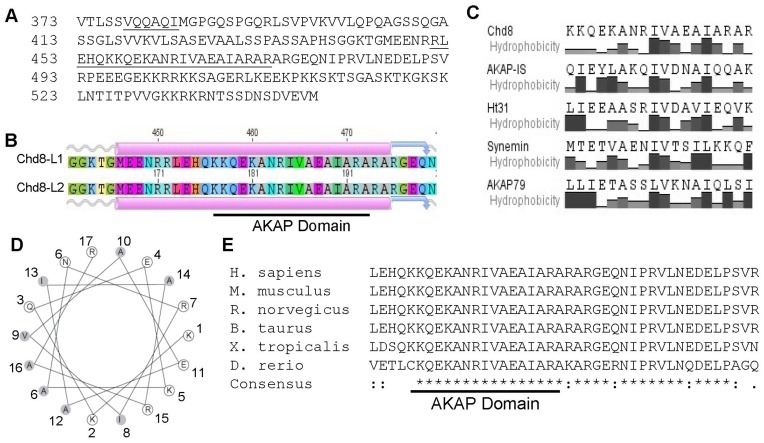
Bioinformatics analysis of Chd8 reveals a predicted α-helix. Bioinformatics analyses of the Chd8 peptide identified in T7 phage display with JPRED2 (A) and Geneious (B) bioinformatics tools show predicted α-helices within the amino acid sequence. C) ClustalW alignment of Chd8 with known AKAP domains. Hydrophobicity plots for each peptide are listed below the amino acid sequence. D) A 2D helical wheel plot was generated for the predicted AKAP domain of Chd8. Hydrophobic amino acids are shaded in gray, and amino acids are numbered starting from the amino terminus. E) Alignment of the predicted AKAP domain (underlined) in Chd8 shows a high level of conservation between species. Sequences from *H. sapiens* (NP_001164100.1), *M. musculus* (NP_963999.2), *R. norvegicus* (NP_075222.2), *B. taurus* (NP_001179063.1), *X. tropicalis* (NP_001131089.2), and *D. rerio* (NP_001189381.1) were used. An asterisk (*) denotes a conserved amino acid, a colon (:) denotes strongly similar amino acids, and a period (.) denotes weakly similar amino acids.

Given the importance of an amphipathic α-helix to the RII:AKAP interaction [24], we used bioinformatics to analyze the predicted secondary structure of Chd8 to identify putative amphipathic helices. We translated the Chd8 cDNA and used this amino acid sequence to predict regions of α-helical domains within the Chd8 sequence. (***Figure 2A***) A search was conducted using GeneiousPro, a bioinformatic platform that includes a tool for generating secondary structure predictions via the EMBOSS Garnier algorithm. [49], [50] A second tool, JPRED, predicts secondary structure and utilizes position-specific scoring matrices, Hidden Markov Model profiles, and structures stored in databases like UniProt and PDB to predict protein structure and accessibility of amino acid residues. [51] Both algorithms predicted an α-helix within the Chd8 peptide that was isolated by the phage display, generating a targeted prediction of an AKAP domain. (***Figure 2A, 2B***).

A ClustalW alignment [52] was performed with the Chd8 fragment and the RII binding domains of several known AKAPs. (***Figure 2C***) The AKAP domains aligned with amino acids within the Chd8 α-helix (455–473aa of Chd8-L1). Hydrophobic profiles of aligned sequences were also similar. (***Figure 2C***) A two dimensional helical wheel plot was generated of the proposed Chd8 RIIα binding site (KKQEKANRIVAEAIARAR). The 2D plot shows clustering of hydrophobic residues on one side of the helix, consistent with RII binding domains of other AKAPs. [53] (***Figure 2D***) This region of the Chd8 sequence is very highly conserved across species. (***Figure 2E***) Thus, using a large scale screening method in conjunction with bioinformatics-based approaches, we identified an amphipathic α-helical structure within the Chd8 peptide fragment that associated with RIIα in our phage display assay. This α-helical region displayed similar amino acid properties as other known AKAPs.

### Chd8 Binds to RIIα *in vitro*

**Figure 3:**
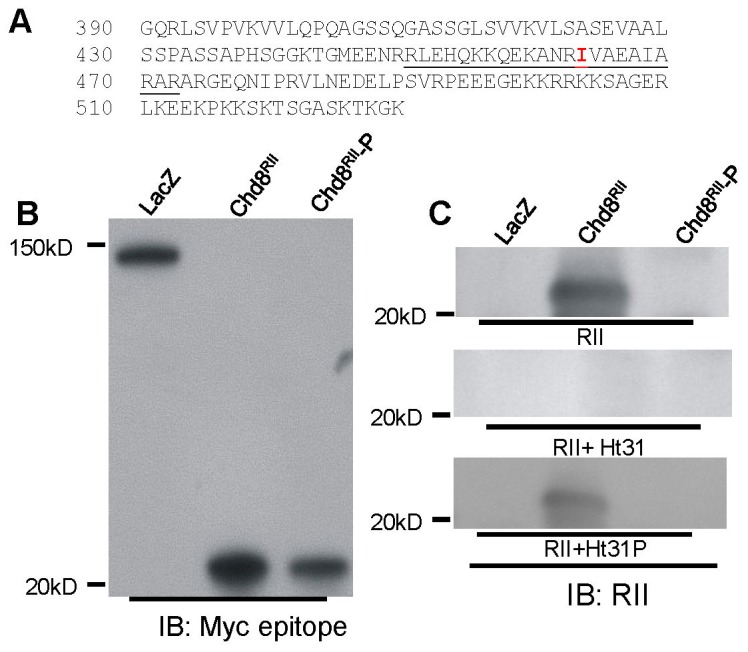
RIIα binds to Chd8^RII^ in RII overlay, but not to Chd8^RII^-P. A) PCR amplification of rat Chd8-S generated a construct with the predicted protein sequence of Chd8, as well as a c-terminal myc tag (not shown). The AKAP domain is underlined. Site-directed mutagenesis was used to mutate I464 (red) to a Pro for the Chd8^RII^-P construct. B) Expression of constructs following induction with IPTG. Constructs were expressed in *E. coli* and grown to log phase before the addition of IPTG to induce expression. As a control for induction of protein, a myc-tagged LacZ construct was used (left lane, 140 kDa MW). Chd8^RII^ (second lane) and Chd8^RII^-P (third lane) were expressed at the predicted molecular weight of approximately 22 kDa. C) RIIα overlay was conducted with Western blots of *E. coli* lysate from bacteria expressing inducible constructs. *Top:* In membranes incubated with RIIα, RIIα/β antibody detected bound RIIα to the lane expressing Chd8^RII^, but not to the lane expressing Chd8^RII^-P. *Middle*: Pre-incubation of RIIα with Ht31, an inhibitor of RII:AKAP interaction, resulted in loss of binding to Chd8^RII^. *Bottom*: Pre-incubation of RIIα with Ht31P, a prolinated form of Ht31 unable to bind RII, did not prevent binding of RIIα to protein in the Chd8^RII^ lane. No corresponding bands were observed in the negative control (LacZ) lanes.

RII overlay was used next to determine the binding capability of RIIα to this fragment of Chd8. RII overlay is frequently used to identify novel PKA binding proteins [46], [54] and exploits the ability of RII to bind AKAP protein on a Western blot. We cloned a 150 amino acid fragment of Chd8 (Chd8^RII^, corresponding to 390–530aa of Chd8-L1). This peptide was then expressed as an inducible His/myc fusion protein in *E. coli*. (***Figure 3A***) Mutation of a hydrophobic residue within the amino acid sequence of an AKAP to a Pro residue is sufficient to abolish α-helical structure and disrupt PKA binding [25], [55], [56]; therefore we used site-directed mutagenesis to introduce a Pro mutation at Ile 464 (Chd8^RII^-P). Based on the bioinformatics analysis of the predicted RIIα binding domain, we predicted that this mutation would eliminate RIIα binding. Immunoblotting methods were used to demonstrate expression of each construct (∼22 kDa MW). (***Figure 3B***).

In RII overlay assays, purified recombinant RIIα bound to a 22 kDa band in protein extracted from bacteria expressing Chd8^RII^, indicative of a 22 kDa RIIα binding protein. (***Figure 3C***, top, center lane) In contrast, no corresponding bound RIIα was detected in lysate from bacteria expressing Chd8^RII^-P, indicating a loss of RIIα binding ability in the fragment carrying the I464P mutation. We then pre-incubated RIIα with Ht31 or Ht31P. Ht31 is a peptide derived from the AKAP domain of AKAP-Lbc that is commonly used to inhibit AKAP:PKA interaction and Ht31P is the same peptide containing a Pro mutation that disrupts the structure of the AKAP domain and prevents association of the peptide with the RII dimer. [24], [25], [57], [58] Pre-incubation of RIIα with Ht31 peptide prevented the binding of RIIα to Chd8^RII^ in RII overlay, whereas the pre-incubation of RIIα with Ht31P had no effect. (***Figure 3C***, center and bottom) We concluded that, like other known AKAPs, RIIα binds Chd8^RII^, but not Chd8^RII^-P, in RII overlay assays.

**Figure 4:**
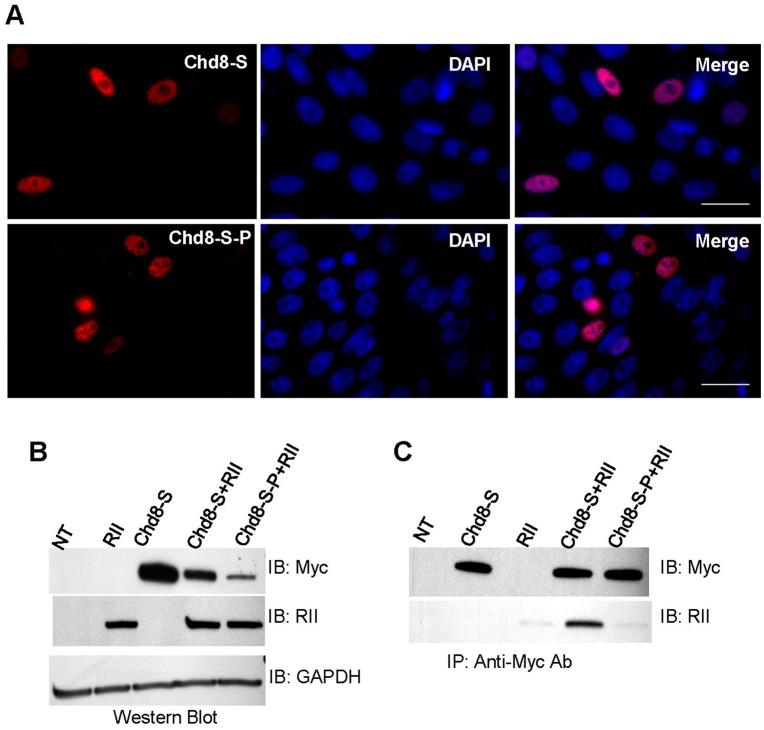
RIIα co-immunoprecipitates with Chd8-S, but not Chd8-S-P. A) Immunofluorescence of transfected cells shows nuclear localization of Chd8-S and Chd8-S-P constructs (red). Cells were imaged with inverted fluorescent microscopy at a magnification of 90X. Scale bar represents 25 µm. B) Western blot analysis of protein extracted from CHO cells transfected with Chd8-S, RIIα, or a combination of Chd8-S and RIIα or Chd8-S-P and RIIα. Chd8 constructs were detected by means of an antibody to a myc epitope tag. RIIα constructs were detected with a pan-RII antibody. GAPDH was used as a loading control. C) Cell lysate for single and co-transfections was subject to immunoprecipitation for myc-tagged constructs. In the single transfection of Chd8-S, immunoprecipitation with antibodies to the myc tag isolated Chd8-S. No product was observed in the single transfection with RIIα. For co-transfections, immunoblotting showed immunoprecipitation of RIIα with Chd8-S, but not Chd8-S-P. No target proteins were identified in immunoprecipitate from untransfected cells (NT). (n = 3, representative blots shown).

We then investigated whether Chd8 and RIIα interact in intact cells. CHO cells were transfected with either Chd8-S or Chd8-S-P, the latter of which contained the same I464P mutation that was sufficient to prevent binding of RIIα to Chd8^RII^-P in the RII overlay assays. Immunofluorescence and Western blotting methods showed that the I464P mutation did not interfere with localization or expression of myc-tagged Chd8-S. (***Figure 4A, 4B***) CHO cells were cotransfected with RIIα and with either Chd8-S or Chd8-S-P. RIIα co-immunoprecipitated with Chd8-S, but not with Chd8-S-P, demonstrating that the I464P mutation in the AKAP domain of Chd8-S resulted in loss of binding to RIIα. (***Figure 4C***) No RIIα was detected in immunoprecipitation of single transfections. We concluded that Chd8-S and RIIα coimmunoprecipiate when coexpressed in CHO cells, but that mutation of the RIIα binding domain in Chd8-S leads to loss of interaction. As I464P was sufficient to prevent coimmunoprecipitation of Chd8-S and RIIα, we also concluded that the RIIα binding domain which we identified by bioinformatics and RIIα overlay is the only RIIα binding domain in Chd8-S.

**Figure 5:**
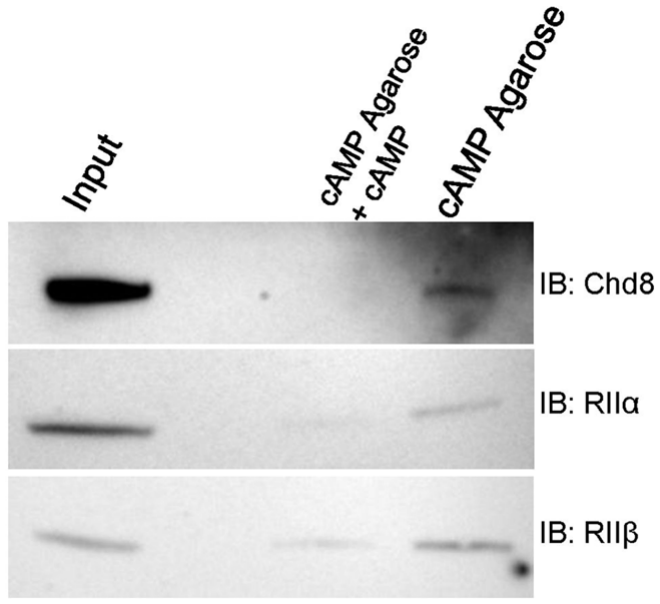
Chd8 is detected with RII subunits in cAMP-pull down. Protein extracted from HEK 293T cells was used for pulldown with cAMP-coupled agarose. Bound proteins were eluted and analyzed by Western blot. *Top:* Chd8 was detected in input lane (left lane) and with eluate from cAMP agarose. The cAMP analogue 8-Br-cAMP was used as a negative control, to compete binding to the cAMP-agarose. Chd8 was not detected in the negative control lane. *Middle:* Detection of RIIα with an RIIα monoclonal antibody showed RIIα in the input lane and the cAMP-agarose lane. *Bottom:* Detection of RIIβ with an RIIβ monoclonal antibody showed RIIβ in the input lane and cAMP-agarose lane. Addition of cAMP reduced, but did not entirely eliminate, pulldown of RIIα and RIIβ in this experiment. (n = 3, representative blots shown).

As experimental and modeling evidence has defined a nuclear microdomain of AKAP-bound PKA in the nuclei of HEK cells [41], we used cAMP-coupled agarose beads to pull down cAMP-binding proteins in HEK cell lysate. RIIα and RIIβ were detected in proteins that eluted with cAMP agarose. Addition of 8-Br-cAMP, a non-hydrolyzable analogue of cAMP, competed with the cAMP agarose for cAMP binding and reduced the amount of RIIα and RIIß pulled down by the beads. A high molecular weight band corresponding with Chd8-L1 was also detected in pulldown assays with cAMP beads, but not in samples incubated with 8-Br-cAMP. (***Figure 5***) We concluded that Chd8 co-elutes with the cAMP-binding proteins RIIα and RIIβ.

### Phosphorylation of RIIα at Serine 96 Inhibits PKA Anchoring by Chd8-S

Autophosphorylation of RIIα by the C subunit at a Ser (Ser96) in the inhibitory domain of RIIα promotes the activation of C for target phosphorylation. [59]–[61] Our recent findings demonstrate that dephosphorylation of Ser96 promotes reassembly of the PKA holoenzyme and reduces binding of RIIα to AKAP15/18. [19], [20] Thus, autophosphorylation of RIIα at Ser96 plays a key role in modulation of PKA activity and localization of the holoenzyme. We next investigated whether RIIα regulation via autophosphorylation affects the interaction between Chd8-S and RIIα.

**Figure 6:**
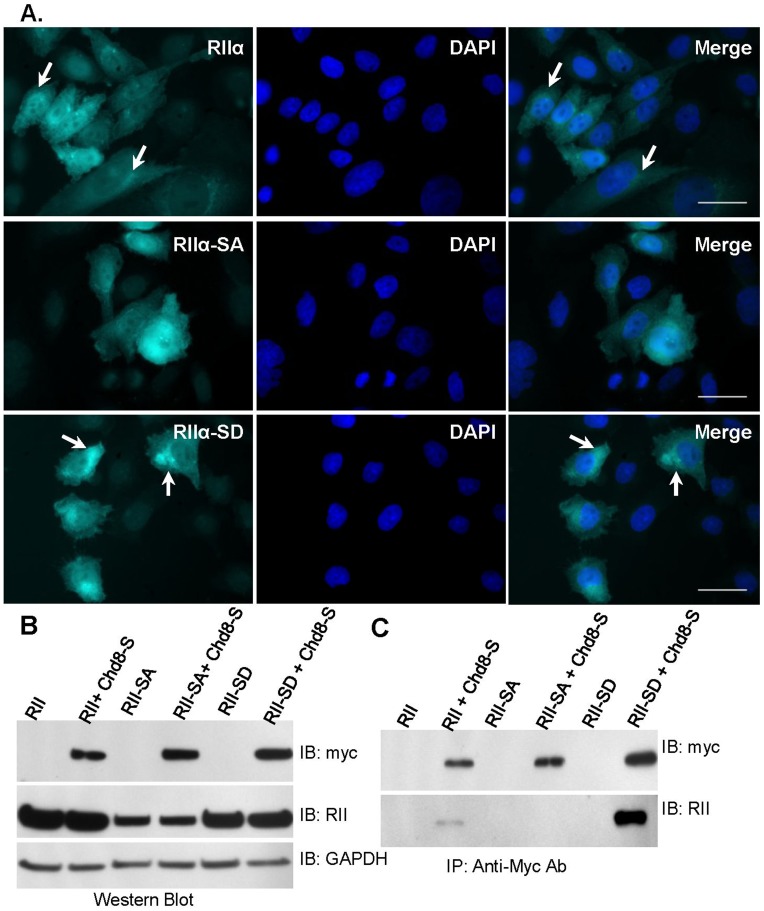
Chd8-S coimmunoprecipitates with RIIα and RIIα-SD, but not RIIα-SA. A) RIIα, RIIα-SA, and RIIα-SD constructs were stably expressed in in CHO cells and visualized through a carboxyl CFP tag. Arrows point to a punctate distribution of the RIIα constructs observed in RIIα and RIIα-SD cell lines. Cells were imaged with inverted fluorescence microscopy and images taken at 90X magnification. Scale bar represents 25 µm. B) Western blot analysis of CHO cells stably expressing RIIα, RIIα-SA, or RIIα-SD alone, or transiently transfected with myc-tagged Chd8-S. Chd8 constructs were detected by means of an antibody to a myc epitope tag. RIIα constructs were detected with a pan-RII antibody. GAPDH was used as a loading control. C) CHO cell lysate was subject to immunoprecipitation with antibodies to the myc tag of Chd8-S. In co-transfected lanes, immunoblot of immunoprecipitate detected RIIα-SD and RIIα in immunoprecipitates of Chd8-S, but not RIIα-SA. No target proteins were detected in the CHO cell lysates expressing RIIα constructs alone. (n = 3, representative blots shown).

We created three CHO cell lines stably expressing RIIα constructs in which Ser96 was not altered (RII), or was mutated to Ala, in order to mimic constitutively dephosphorylated RIIα (RII-SA). RIIa was also mutated to Asp to mimic constitutively phosphorylated RIIα (RII-SD). Expression of each RIIα construct was verified by immunofluorescence, using a CFP tag (***Figure 6A***), and by Western blot analysis (***Figure 6B***). Each cell line was transiently transfected with Chd8-S. (***Figure 6B***) Chd8-S was immunoprecipitated from all transfected cultures. Western blot analysis of the Chd8-S immunoprecipitate identified RII and RII-SD, but not RII-SA. (***Figure 6C***) As this result was consistent with the anchoring dynamics of other AKAPs [19], [20], we concluded that dephosphorylation of Ser96 (RII-SA) eliminates the interaction of RIIα and Chd8-S, whereas autophosphorylation of RIIα at Ser96 promotes binding of Chd8 and RIIα.

### Subcellular Localization of RII and Chd8

Consistent with the first study of Chd8-S [37], [38], our localization studies of cells overexpressing the Chd8-S isoform show that it is restricted to the nucleus (***Figure 4A***). [37], [38] However, since those studies were published, additional isoforms of Chd8 (Chd8-L1 and Chd8-L2) have been described. Other AKAP genes, including those for AKAP350 [62] and AKAP-Lbc [63], [64] encode multiple isoforms with different patterns of subcellular localization. To determine if the longer Chd8 isoforms exhibit the same subcellular localization pattern, we examined the endogenous localization of Chd8.

**Figure 7:**
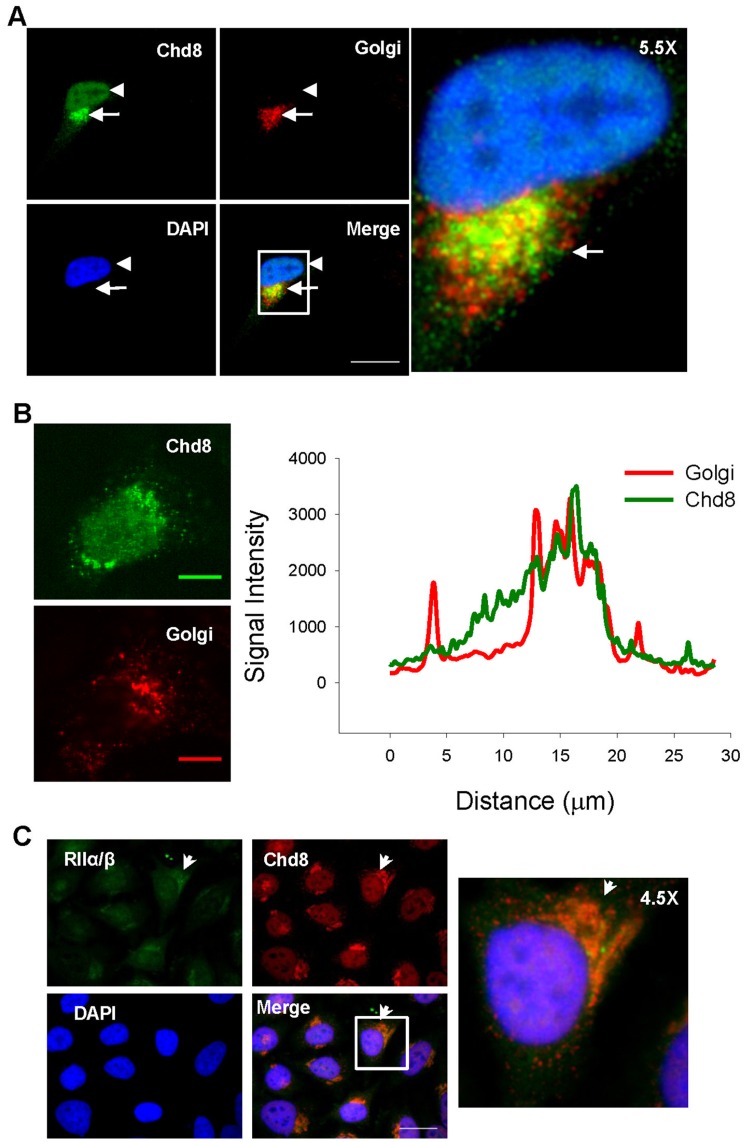
Subcellular localization of Chd8 and RIIα/β in HeLa cells. A) Immunofluorescence of endogenous Chd8 (green) identified nuclear Chd8, and also a discrete perinuclear staining (arrow, arrowhead). Immunofluorescence of the Golgi apparatus (red) with an antibody to human Golgi reveals distinct perinuclear localization. Merge shows that the perinuclear pool of Chd8 is in close proximity (arrowhead) to or overlapping with (arrow) the Golgi apparatus. Inset shows cell that was magnified 5.5× for the right panel. Cells were imaged with inverted fluorescence microscopy and images taken at 90× magnification. Scale bar represents 25 µm. B) Confocal microscopy of Chd8 (green) and Golgi (red) immunofluorescence in the same transverse slice. The graph represents the plot profile for signals across each channel in the same plane. Images taken at 63× magnification, the scale bar represents 10 µm. C) Costaining for RII (green) and Chd8 (red) in HeLa cells. The merge reveals overlapping signals between RII and Chd8 in the perinuclear staining (arrows). Inset shows cell that was magnified 4.5X for the right panel. Cells were imaged with inverted fluorescence microscopy and images taken at 90X magnification. Scale bar represents 25 µm.

We immunostained HeLa human adenocarcinoma cells, which have been previously used to identify Chd8 binding partners [31], with a polyclonal antibody raised against a 50 amino acid fragment of the carboxy terminus of Chd8-L1 and Chd8-L2. Our immunostaining of endogenous Chd8 revealed nuclear staining (***Figure 7A***, arrowhead; Figure S2A), but, interestingly, we also found a perinuclear pattern of immunofluorescence. (***Figure 7A*** ***, arrows***) This staining pattern was reproduced using an alternate antibody raised against the amino terminus of Chd8. (Figure S4A) Because of the distinctive perinuclear pattern, we costained HeLa cells with an antibody raised against the Golgi fraction of human cells. We observed perinuclear immune-labeling of Chd8 in the region of the Golgi apparatus. (***Figure 7A***) Confocal microscopy of HeLa cells co-labeled with antibodies raised against Chd8 and Golgi showed similar plot profiles of the immunofluorescence patterns in the same focal plane. (***Figure 7B***) Addition of the immunogen against which the Chd8 immunostaining antibody was raised resulted in loss of both the nuclear and perinuclear immune-labeling. (Figure S2) Costaining of HeLa cells with antibodies against Chd8 and PKA RIIα/β demonstrated perinuclear staining for both proteins. Overlay of images demonstrated subcellular colocalization of these signals, particularly in the perinuclear domain. (***Figure 7C*** ***,*** arrows).

### Chd8 is Expressed in Rat Cardiac Development

Chd8 was isolated in our screen for novel cardiac AKAPs. [27] As Chd8 has been implicated in embryonic development [36], [37], [39], we examined the expression of Chd8 in developing rat cardiac tissue. In a study of mouse embryos, Chd8 mRNA is expressed at high levels early in embryogenesis and lower levels found in adult mice. [37], [39] Given Chd8’s role as a negative regulator of p53, differential regulation of Chd8 may contribute to p53-dependent apoptosis required in organogenesis [36], or to the selective regulation of the Wnt/β-catenin pathway during development [31], [35].

**Figure 8:**
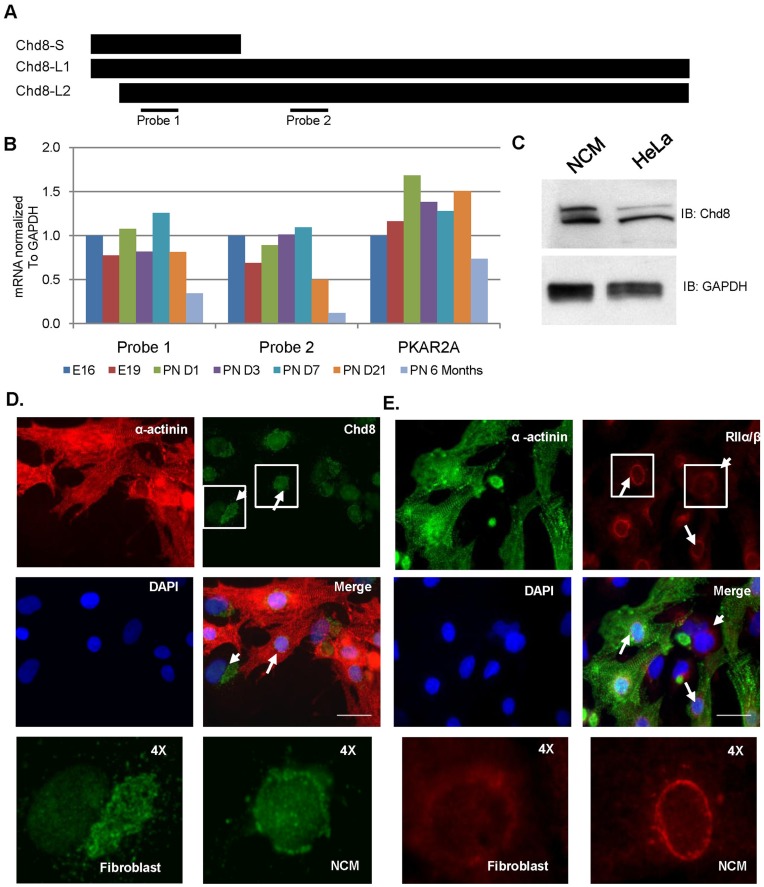
Expression and subcellular localization of Chd8 in cardiac development and in post-natal NCMs. A) A representation of the targets of the two sets of TaqMan probes used to measure Chd8 mRNA. Probe 1 (ABI IDRn00576005_m1) spans exon 2–3 and covers the RII binding domain. Probe 2 (ABI Rn01414467_m1) spans exons 12–13, which encode the helicase domain and detects only the two longest isoforms. B) Relative amounts of mRNA of Chd8 (Probe 1 and Probe 2) and RIIα (PKAR2A), normalized to GAPDH and calculated by the 2^−ΔΔCtt^ method. C) Western blot was used to detect Chd8-L1 and Chd8-L2 in NCMs. HeLa cell lysate was used as a positive control. D) NCMs were fixed at four days in culture and stained for Chd8 (green) and α-actinin (red). Arrows indicate myocytes, while arrowheads indicate fibroblasts. E) NCMs were fixed at four days in culture and stained for α-actinin (green) and RIIα/β (red). Arrows indicate myocytes, while arrowheads indicate fibroblasts. Cells were imaged with inverted fluorescence microscopy and images taken at 90X magnification. Scale bar represents 25 µm.

Two sets of Taqman probes were used to detect Chd8 mRNA in developing cardiac muscle. (***Figure 8A***) Probe 1, which spans exons 2–3 and encompasses the AKAP binding domain of Chd8, recognizes all three isoforms transcripts of Chd8. Probe 2 recognizes Chd8-L1 and Chd8-L2 and spans exons 12 and 13, which encode the helicase domain. A probe against the mRNA transcript for RIIα (PKAR2A) was used to measure RIIα mRNA. We detected high levels of cardiac CHD8 mRNA at PN7, followed by a decrease to low but detectable levels in 6 month-old rat heart. (***Figure 8B***).

We next utilized rat neonatal cardiac myocytes to study expression of Chd8 protein in the heart. Western blot analysis of myocyte protein extract identified both the Chd8-L1 and Chd8-L2 isoforms. (***Figure 8C***) Immunofluorescence of fixed cultures showed Chd8 expression in both myocytes (***Figure 8D***, arrows) and fibroblasts (***Figure 8E***, arrowheads). As we previously reported above for HeLa cells, the antibody to Chd8 detected nuclear immunostaining, as well as a perinuclear pool of Chd8, in both cell types. (***Figure 8D***) This staining pattern was reproduced using an alternate antibody raised against the amino terminus of Chd8. (Figure S4B) In contrast, we observed only perinuclear staining of RIIα/β in myocytes and fibroblasts. (***Figure 8E***) These findings indicated that Chd8 is expressed in cardiac cells, where it localizes to both nuclear and perinuclear domains, similar to the immunostaining pattern seen in HeLa cells. (Figure S2B).

## Discussion

We have shown that Chd8, previously identified as a chromatin binding protein, also binds RIIα in intact cells and is thus a new member of the AKAP family. Similar to our previous findings that phosporylated RIIα has a higher affinity for AKAPs than dephosphorylated RIIα [19], [20], we demonstrated that phosphorylation of RIIα is required for Chd8:PKA association. Furthermore, in addition to the identified nuclear localization of Chd8, we also demonstrated a novel perinuclear localization of Chd8 in close proximity to the Golgi apparatus.

### Chd8 Contains an AKAP Domain in its Amino Terminus

Having established RIIα binding to Chd8 by phage display, we used bioinformatics, including the prediction of secondary structure, predictive modeling, alignments, to identify the AKAP domain of Chd8. (***Figure 2***) Bioinformatics has been previously used to study R:AKAP interactions, and is a powerful tool when paired with experimental approaches. [58], [65], [66] A study by McLaughlin *et al*, published while this manuscript was in progress, also identified a 24 amino acid peptide of Chd8 that overlaps with the Chd8 PKA binding domain. This domain was identified using our own bioinformatics-based approach. McLaughlin *et al* used a panel of Ala mutations within the Sphingosine kinase interacting protein (SKIP) to show that SKIP is a dual AKAP. [65] In contrast, another recent study used molecular modeling together with site-directed mutagenesis and immunoprecipitation, to show that SKIP is exclusively an RI-specific AKAP that does not bind RII. [66] These divergent findings highlight the importance of pairing experimental manipulation with bioinformatics approaches.

Our findings that dephosphorylation of RIIα at Ser96 prevents the binding of Chd8 and RIIα suggest a mechanism by which anchoring of PKA to Chd8 may be regulated within nuclear and/or perinuclear microdomains. (***Figure 5***) RIIα phosphorylation at different sites modulates the affinity of the RIIα dimer for AKAPs within the same subcellular compartment. [10], [67] Our previous work shows that PKA autophosphorylation of RIIα in the inhibitory domain at Ser96 increases the affinity of RIIα for AKAP15/18. [20] Past work from our lab also demonstrated that the relative decrease in affinity of RIIα for AKAPs upon dephosphorylation at Ser96 varies between AKAPs. For example, the decrease in binding affinity between dephosphorylated RIIα and AKAP15/18 is more than 600 times greater than the decrease in binding affinity for dephosphorylated RIIα and AKAP-Lbc, as compared to phosphorylated RIIα. [16], [19] Our studies likewise demonstrate that phosphorylation of RIIα at Ser96 increases the probability of RIIα association with Chd8. In unstimulated cells, low levels of RIIα phosphorylation have been observed, under baseline conditions, and exogenously expressed RIIα can be phosphorylated and dephosphorylated at Ser96. [20] Since we observed immunoprecipitation of Chd8-S with RII-SD but not RII-SA, it is likely that the RIIα that is immunoprecipitated in our assay is phosphorylated. We also observed a perinuclear distribution of RII and RII-SD. (***Figure 6B***, arrows) We hypothesize that phosphorylation of RIIα at Ser96 may serve as a molecular switch that increases binding affinity for Chd8 versus RIIa binding to other AKAPs in the same compartment.

AKAPs target other proteins that regulate stability of cAMP and the phosphorylation state of R. [68] Based on our findings, we propose that Chd8 anchors PKA in close proximity to p53, histone H1, or β-catenin in the nucleus following activation of the PKA signaling pathway. (***Figure 1***) Other AKAPs bind phosphodiesterases or protein phosphatases, which attenuate the PKA pathway after the elevation of cAMP. [3], [68] Recent work characterizing a nuclear PKA microdomain identified candidate binding proteins for nuclear AKAPs, including soluble adenylyl cyclase (AC). Soluble AC could participate in nuclear PKA signaling. [42], [69] One intriguing result arose from modeling the activation of a nuclear microdomain of cAMP and PKA. [41] This study reported a nuclear microdomain of PKA that permitted rapid activation kinetics of PKA in the nucleus following activation of sAC. Introduction of a hypothetical nuclear AKAP into this kinetic model likewise implicated the importance of PKA and phosphodiesterase anchoring in the nucleus, although no specific AKAP was manipulated experimentally. [41] Chd8 is a possible candidate for this unknown AKAP.

### Chd8 Exists within Nuclear and Perinuclear Microdomains

Few AKAPs have been reported to reside in the nucleus. Despite a longstanding model in which C subunits of PKA translocate to the nucleus following elevation of cAMP, several reports indicate that a nuclear microdomain of PKA [41], [42], [70]–[73] and cAMP [42], [69], [74] does exist, possibly governed by sAC. [41], [42], [69], [75] The nuclear distribution of PKA regulatory subunits has been reported in multiple cell lines and tissues [41], [70], [72], [73], [76]–[79], including HeLa cells [42]. Localization of AKAPs to the nucleus permits rapid and effective signal transduction in the nuclear compartment. [41] To date, nuclear AKAP95 has been best characterized: PKA anchoring via AKAP95 is required for proper chromatin condensation during mitosis. [67], [80]–[82] AKAP7 [83] and nAKAP150 [76] localize to the nucleus during development, whereas the splicing factor SFRS17A is a dual AKAP that regulates pre-mRNA splicing. [84] Our identification of an AKAP domain in Chd8 expands the understanding of the roles of nuclear AKAPs.

Some AKAPs, e.g. AKAP350, AKAP13, exhibit alternative subcellular localization of different isoforms. [62]–[64] Our results indicate that Chd8 exists in at least two microdomains, one nuclear and one perinuclear. (***Figure 7***) It remains to be determined if the two pools of Chd8 contain different isoforms, or if Chd8-L1 and Chd8-L2 are present in both. Given the functional diversity of AKAPs, isoforms of Chd8 may play differential role in anchoring PKA to different subcellular microdomains.

Similar to its subcellular distribution in HeLa cells, we showed that Chd8 exhibits nuclear and perinuclear localization in cardiac cells. (***Figure 8D***) The distribution of the perinuclear immunostaining in cardiac myocytes differed from the distribution in fibroblasts. Interestingly, in myocytes, connexin-43, which is trafficked from the Golgi apparatus to cell junctions by anterograde vesicular transport, exhibits a similar compact perinuclear immunostaining pattern, attributed to its localization in the Golgi. [85] The similarity of staining patterns suggests colocalization of perinuclear Chd8 with the Golgi apparatus in myocytes. Our immunostaining of RII in myocytes did not show detectable RII in the nuclei of myocytes or cardiac fibroblasts.(***Figure 8E***) As analysis of mouse heart protein has shown expression of all four isoforms of R subunit [86], it is possible that nuclear localization of R varies between cell types, or that rat heart expresses PKA isoforms in a different pattern than in murine cardiac tissue. Alternatively, the nuclear microdomain of PKA may be more easily detectable in other cell lines. HeLa cells, among other cell types, have been shown to contain nuclear PKA holoenzyme. [41], [42], [78] Thus, Chd8 may act as an AKAP in the nucleus and in the perinuclear domain of HeLa cells, whereas in cardiac myocytes, PKA anchoring by Chd8 may be restricted to the perinuclear domain.

The mechanism by which Chd8 localizes to the perinuclear region remains to be determined. The phosphorylation of residues within an NLS is one mechanism to regulate nuclear localization of a protein. [87] Analysis of the Chd8 NLS with PKAps, a program designed to predict PKA phosphorylation sites, identifies several potential PKA targets in the Chd8 NLS. [88] (Table S1) Given the close proximity of anchored PKA to the NLS, one function of anchored PKA may be the phosphorylation of Chd8 itself.

### Chd8 is Expressed at High Levels in Post-natal Heart

We demonstrated that Chd8 is expressed during embryonic and post-natal cardiac development and also in myocytes and fibroblasts from post-natal rat hearts. (***Figure 8***) Previous reports described peak levels of Chd8 mRNA in whole mouse embryos, with a decline of Chd8 mRNA in newborn mice. [37] In contrast, our results indicate that, in rat heart, a high level of Chd8 mRNA is detected for at least a week after birth. To date, Chd8, a regulator of cell cycle genes and apoptosis, has been studied in cancer cell lines and in vascular smooth muscle cells, capable of division in culture. In contrast, cardiac myocytes grow in three phases: a fetal period characterized by proliferative hyperplasia, a perinatal phase between birth and weaning that is characterized by hypertrophic growth and binucleation, and a third phase that spans weaning through adulthood, where myocytes grow primarily by hypertrophy. [89] The immediate postnatal period is a time of intense cardiac remodeling. [89], [90] A study of sheep heart reported that right ventricle mass is greater than the left *in utero*, This imbalance was reversed in the weeks following birth as left ventricular cardiac myocytes grew larger. [90] A large scale analysis of rat cardiac DNA, RNA, and protein in the three stages of development also showed an oscillation of ventricular DNA in the perinatal period, with the highest recorded time point at PN7. [89] Given the role of Chd8 in regulating genes that correspond with cell growth and survival, the high levels of Chd8 mRNA expression observed at PN7 raise the possibility that elevated Chd8 protein plays a role in these transcriptional events.

## Conclusions

In summary, we demonstrated that Chd8 contains an amino terminal RIIα binding domain, between residues 455 and 473, and that this domain is required for RIIα binding to Chd8. Immunofluorescence indicates a non-nuclear pool of Chd8 that appears to colocalize with RII and in proximity to markers against the Golgi apparatus. Nuclear and perinuclear microdomains of Chd8 were also identified in HeLa cells and in isolated rat NCMs. Moreover, dephosphorylation of RIIα at Ser96 eliminates binding of RIIα to Chd8-S, whereas RIIα subunits psuedophosphorylated at Ser96 bind Chd8-S. These results indicate that Chd8 is a novel AKAP and demonstrate roles for Chd8 beyond its regulation of development, transcription, and cell survival.

## Supporting Information

Figure S1 **Negative control immunostaining with secondary antibodies in CHO and HeLa cells.** A) CHO cells were incubated with Alexafluor Goat anti-Mouse 568 and imaged in conjunction with Chd8-S/Chd8-S-P transfections in Figure 4. Cells were imaged with inverted fluorescent microscopy at a magnification of 60X. Scale bar represents 38 µm. B) HeLa cells were incubated with Alexafluor Goat anti-Rabbit 488 and Alexafluor Goat anti-Mouse 568 and imaged with inverted fluorescent microscopy at a magnification of 90X. Pane label indicates 488 or 568 channels. Scale bars represent 25 µm. C) HeLa cells were incubated with Alexafluor Donkey anti-Goat 568 and Alexafluor Donkey anti-Mouse 488 and imaged with inverted fluorescent microscopy at a magnification of 90X. Scale bars represent 25 µm. Pane label indicates 488 or 568 channels. D) HeLa cells were incubated with Alexafluor Donkey anti-Rabbit 568 and Alexafluor Donkey anti-Goat 488 and imaged with inverted fluorescent microscopy at a magnification of 90X. Scale bars represent 25 µm. Pane label indicates 488 or 568 channels.
(TIF)

Figure S2 **Specificity of Chd8 and RIIα/β antibodies in immunofluorescence.** A) *Upper panels:* Unblocked immunofluorescence of Chd8. *Lower panels:* Immunofluorescence of endogenous Chd8 in HeLa cells with antibody preincubated for 1 hour with a three-fold excess of the peptide encompassing the antibody epitopes. Insets show immunofluorescence with secondary antibody (Alexafluor Donkey anti-Rabbit 568) alone. B) *Upper panels:* Unblocked immunofluorescence of RIIα/β. *Lower panels:* Immunofluorescence of endogenous RIIα/β in HeLa cells with antibody preincubated for 1 hour with a three-fold excess of purified recombinant RIIα. Insets show immunofluorescence with secondary antibody (Alexafluor Donkey anti-Goat 488) alone. All cells were imaged with inverted fluorescent microscopy at a magnification of 90X. Scale bars represent 25 µm.
(TIF)

Figure S3 **Negative control immunostaining with secondary antibodies in NCMs.** A) Isolated rat cardiac cells were incubated with Alexafluor Goat anti-Mouse 568 and Alexafluor Goat anti-Rabbit 488 and imaged with inverted fluorescent microscopy at a magnification of 90X. B) Isolated rat cardiac cells were incubated with Alexafluor Donkey anti-Goat 568 and Alexafluor Donkey anti-Mouse 488 and imaged with inverted fluorescent microscopy at a magnification of 90X. Scale bars represent 25 µm.
(TIF)

Figure S4 **Immunofluorescence of HeLa cells and NCM with an alternate Chd8 antibody.** A) *Top Row*: HeLa cells were incubated with Chd8-Sigma antibody and Alexafluor Goat anti-Rabbit 488, and imaged with inverted fluorescent microscopy. *Bottom Row*: HeLa cells were incubated with secondary antibody alone. Scale bars represent 25 µm. B) NCMs were fixed at four days in culture and stained for α-actinin (red) and Chd8-Sigma (green), detected with Alexafluor Goat anti-mouse 568 and Alexafluor Goat anti-rabbit 488, respectively.Short arrows indicate myocytes, while long arrows indicate fibroblasts. Cells were imaged with inverted fluorescence microscopy. Scale bar represents 25 µm.
(TIF)

Table S1 **Prediction of phosphorylation sites on the amino terminus of Chd8.** The prediction program PKAps was used to generate predictions of PKA phosphorylation targets in the first 800 amino acids of Chd8 [88]. Phosphorylation sites that occur within a known domain of Chd8 are marked. Phosphorylation sites that fall within regions. The phosphorylated residue is bolded and underlined.
(DOC)
