# Supplementary figures and images for: Chromodomain Helicase Binding Protein 8 (Chd8) Is a Novel A-Kinase Anchoring Protein Expressed during Rat Cardiac Development
Source: PLoS One. 2012 Oct 10;7(10):e46316. doi: 10.1371/journal.pone.0046316 (PMC3468582; doi:10.1371/journal.pone.0046316)

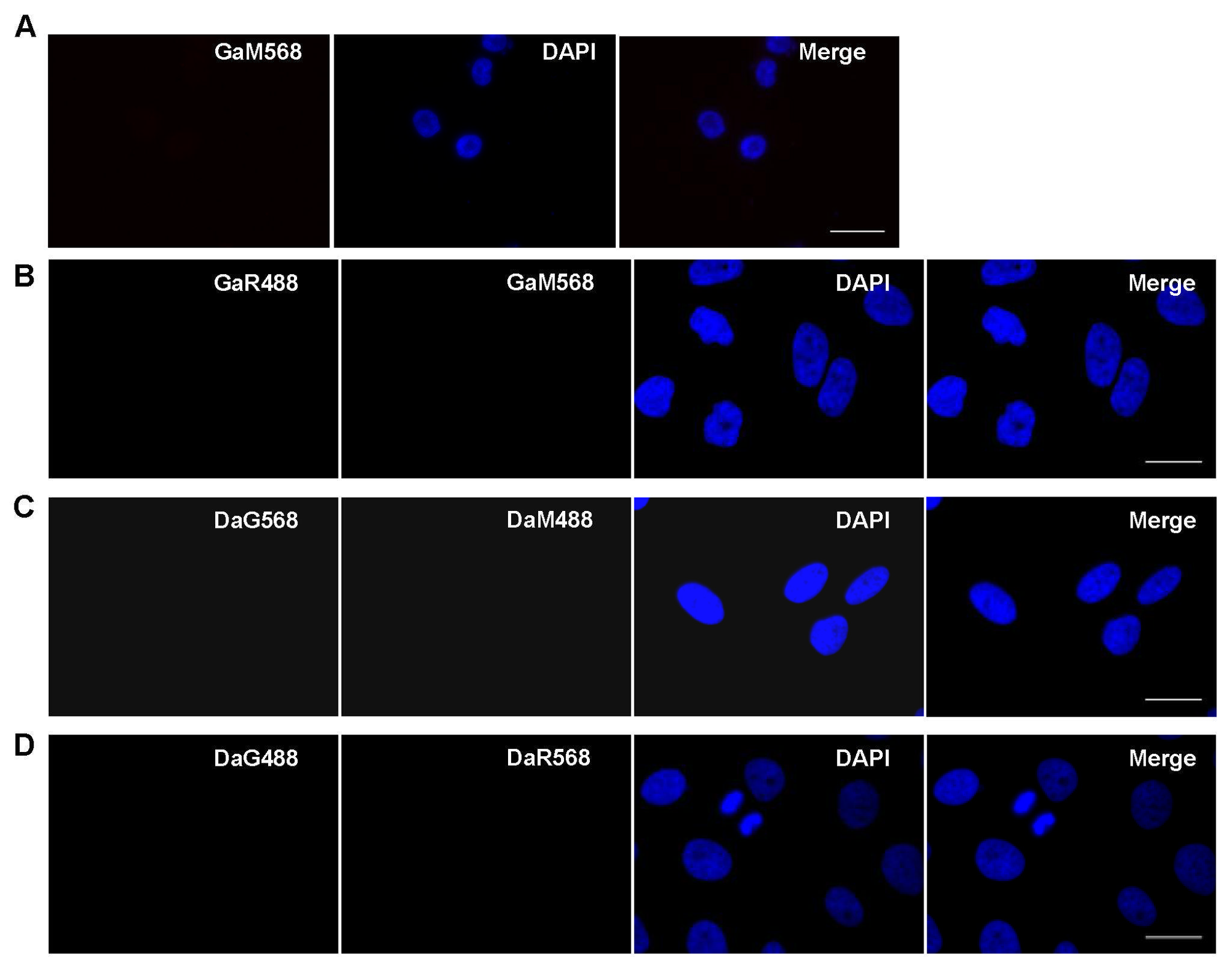

Supplement: Figure S1 — Negative control immunostaining with secondary antibodies in CHO and HeLa cells. A) CHO cells were incubated with Alexafluor Goat anti-Mouse 568 and imaged in conjunction with Chd8-S/Chd8-S-P transfections in Figure 4. Cells were imaged with inverted fluorescent microscopy at a magnification of 60X. Scale bar represents 38 µm. B) HeLa cells were incubated with Alexafluor Goat anti-Rabbit 488 and Alexafluor Goat anti-Mouse 568 and imaged with inverted fluorescent microscopy at a magnification of 90X. Pane label indicates 488 or 568 channels. Scale bars represent 25 µm. C) HeLa cells were incubated with Alexafluor Donkey anti-Goat 568 and Alexafluor Donkey anti-Mouse 488 and imaged with inverted fluorescent microscopy at a magnification of 90X. Scale bars represent 25 µm. Pane label indicates 488 or 568 channels. D) HeLa cells were incubated with Alexafluor Donkey anti-Rabbit 568 and Alexafluor Donkey anti-Goat 488 and imaged with inverted fluorescent microscopy at a magnification of 90X. Scale bars represent 25 µm. Pane label indicates 488 or 568 channels. (TIF) [file pone.0046316.s001.tif]

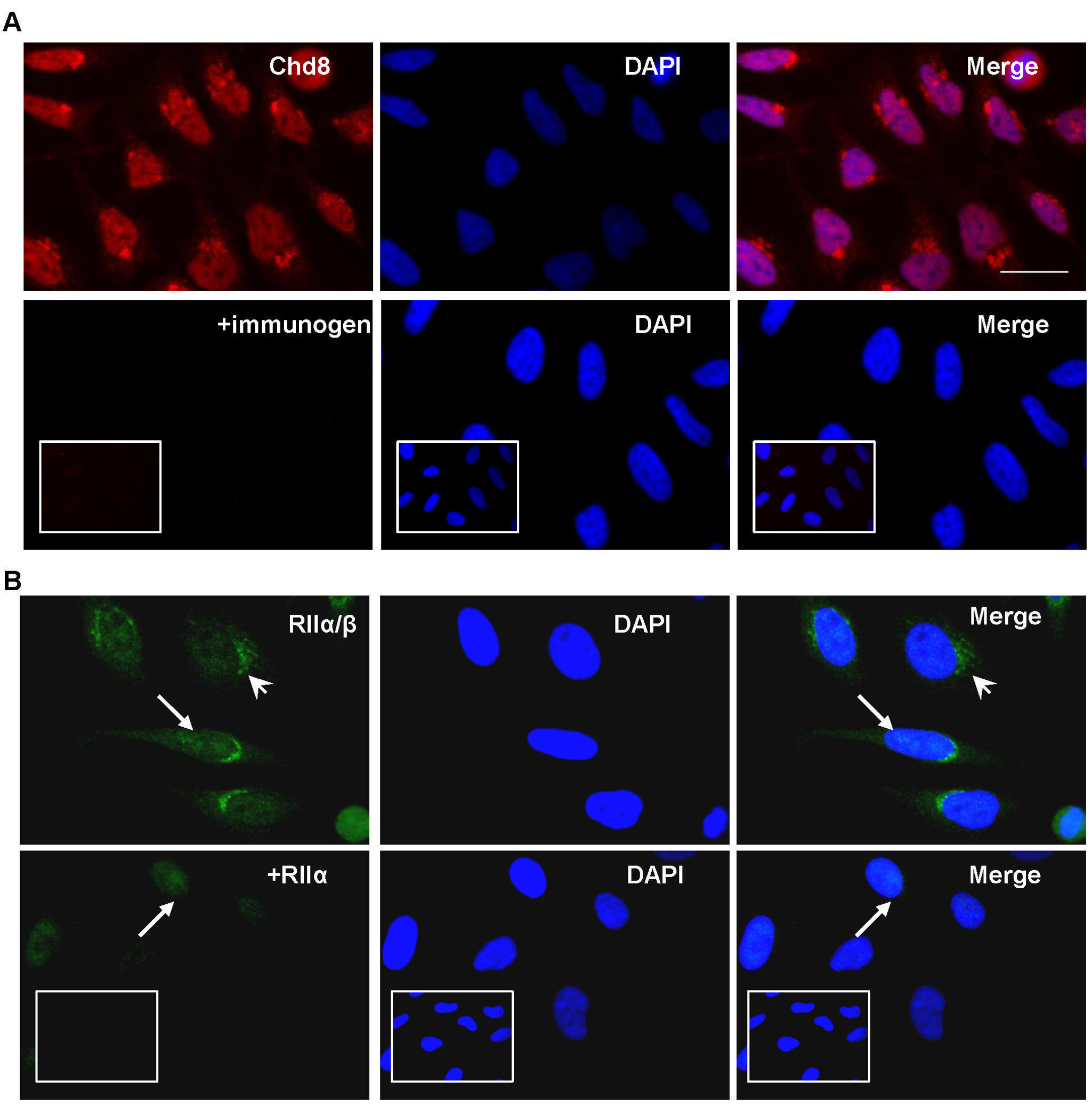

Supplement: Figure S2 — Specificity of Chd8 and RIIα/β antibodies in immunofluorescence. A) Upper panels: Unblocked immunofluorescence of Chd8. Lower panels: Immunofluorescence of endogenous Chd8 in HeLa cells with antibody preincubated for 1 hour with a three-fold excess of the peptide encompassing the antibody epitopes. Insets show immunofluorescence with secondary antibody (Alexafluor Donkey anti-Rabbit 568) alone. B) Upper panels: Unblocked immunofluorescence of RIIα/β. Lower panels: Immunofluorescence of endogenous RIIα/β in HeLa cells with antibody preincubated for 1 hour with a three-fold excess of purified recombinant RIIα. Insets show immunofluorescence with secondary antibody (Alexafluor Donkey anti-Goat 488) alone. All cells were imaged with inverted fluorescent microscopy at a magnification of 90X. Scale bars represent 25 µm. (TIF) [file pone.0046316.s002.tif]

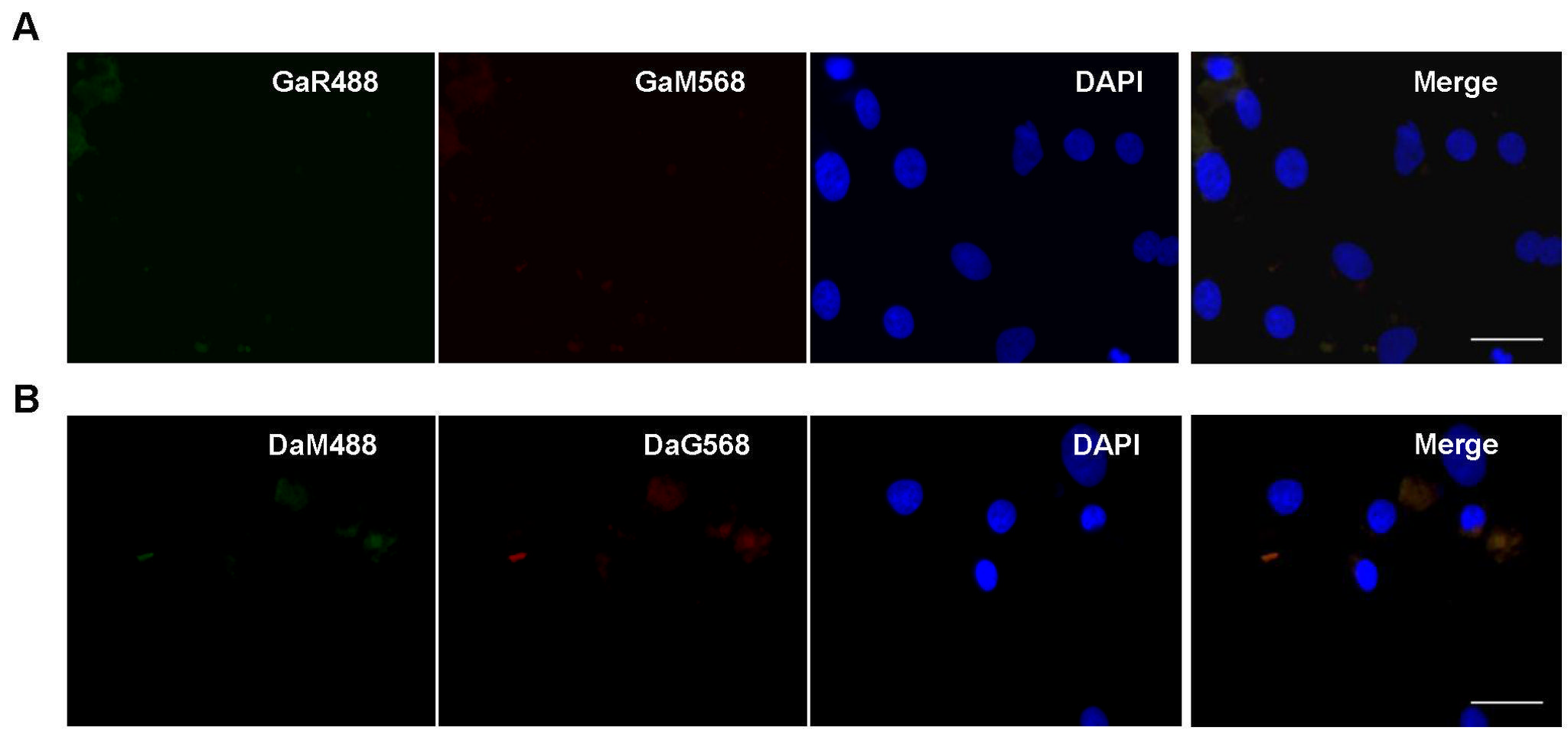

Supplement: Figure S3 — Negative control immunostaining with secondary antibodies in NCMs. A) Isolated rat cardiac cells were incubated with Alexafluor Goat anti-Mouse 568 and Alexafluor Goat anti-Rabbit 488 and imaged with inverted fluorescent microscopy at a magnification of 90X. B) Isolated rat cardiac cells were incubated with Alexafluor Donkey anti-Goat 568 and Alexafluor Donkey anti-Mouse 488 and imaged with inverted fluorescent microscopy at a magnification of 90X. Scale bars represent 25 µm. (TIF) [file pone.0046316.s003.tif]

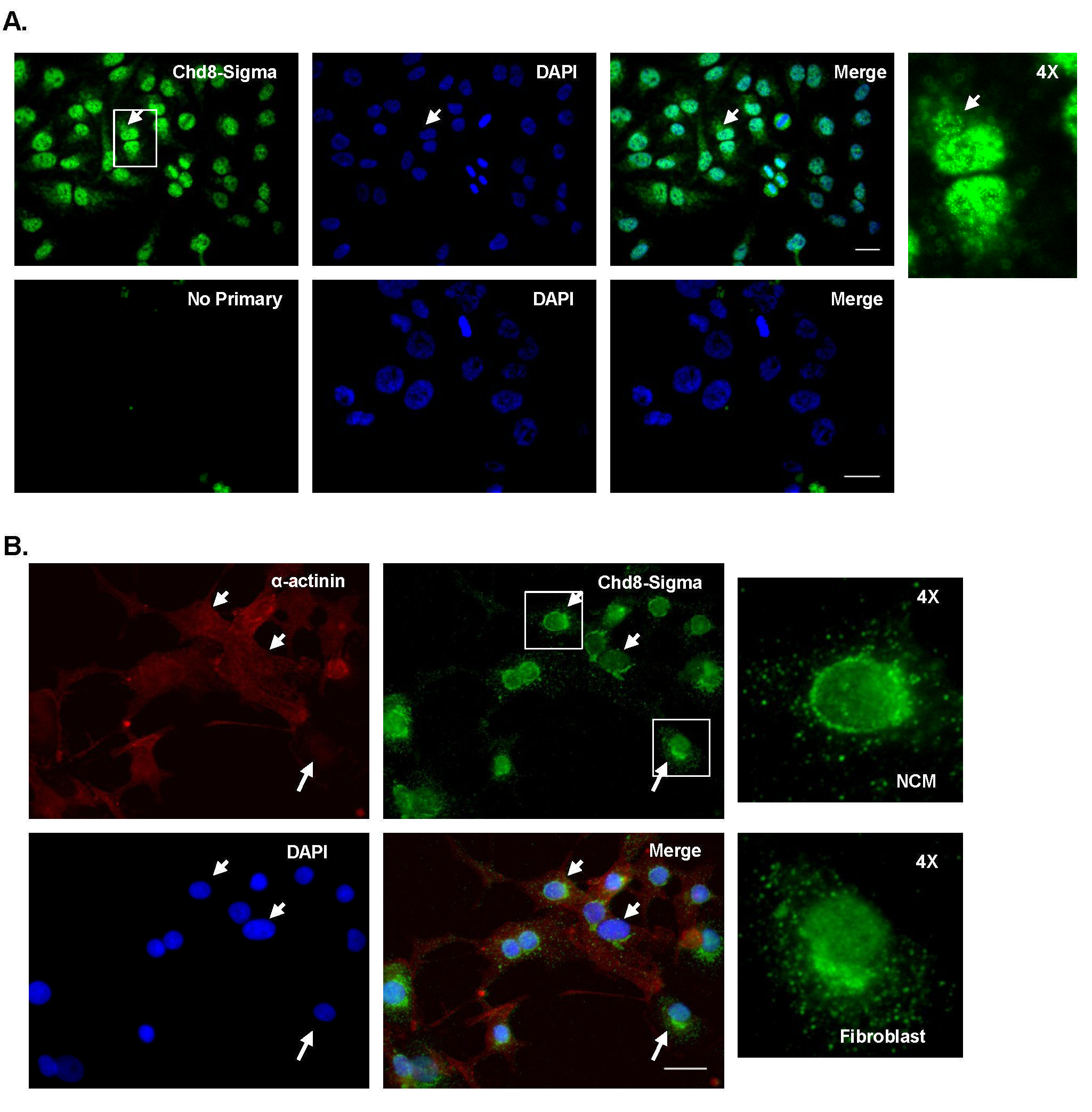

Supplement: Figure S4 — Immunofluorescence of HeLa cells and NCM with an alternate Chd8 antibody. A) Top Row: HeLa cells were incubated with Chd8-Sigma antibody and Alexafluor Goat anti-Rabbit 488, and imaged with inverted fluorescent microscopy. Bottom Row: HeLa cells were incubated with secondary antibody alone. Scale bars represent 25 µm. B) NCMs were fixed at four days in culture and stained for α-actinin (red) and Chd8-Sigma (green), detected with Alexafluor Goat anti-mouse 568 and Alexafluor Goat anti-rabbit 488, respectively.Short arrows indicate myocytes, while long arrows indicate fibroblasts. Cells were imaged with inverted fluorescence microscopy. Scale bar represents 25 µm. (TIF) [file pone.0046316.s004.tif]
