# Supplementary material for: Chromodomain Helicase Binding Protein 8 (Chd8) Is a Novel A-Kinase Anchoring Protein Expressed during Rat Cardiac Development
Source: PLoS One. 2012 Oct 10;7(10):e46316. doi: 10.1371/journal.pone.0046316 (PMC3468582; doi:10.1371/journal.pone.0046316)
Supplement: Table S1 — Prediction of phosphorylation sites on the amino terminus of Chd8. The prediction program PKAps was used to generate predictions of PKA phosphorylation targets in the first 800 amino acids of Chd8 [88]. Phosphorylation sites that occur within a known domain of Chd8 are marked. Phosphorylation sites that fall within regions. The phosphorylated residue is bolded and underlined. (DOC) [file pone.0046316.s005.doc]

**Table S1: Prediction of phosphorylation sites on the amino terminus of Chd8.**

| **Position** | **Sequence** | **Domain** |
| --- | --- | --- |
| 256 | PVKG**S**APAG | - |
| 288 | SKRI**T**LVLQ | - |
| 395 | PGQR**S**VPVK | - |
| 423 | VKVL**S**ASEV | - |
| 506 | RRKK**S**AGER | NLS |
| 519 | KPKK**S**KTSG | NLS |
| 521 | KKSK**T**SGAS | NLS |
| 522 | KSKT**S**GASK | NLS |
| 531 | TKGK**S**KLNT | NLS |
| 535 | SKLN**T**ITPV | NLS |
| 548 | RKRN**T**SSDN | NLS |
| 549 | KRNT**S**SDNS | NLS |
| 576 | QKRR**S**NRQV | H1/β-catenin |
| 586 | RKKY**T**EDLD | H1/β-catenin |
| 703 | KRFK**T**KMAQ | Chromodomain |
| 780 | KRIQ**S**RHPE | - |
